# Ciliary control of adipocyte progenitor cell fate regulates energy storage

**DOI:** 10.3389/fcell.2022.1083372

**Published:** 2022-12-06

**Authors:** Sierra R. Scamfer, Mark D. Lee, Keren I. Hilgendorf

**Affiliations:** Department of Biochemistry, University of Utah School of Medicine, Salt Lake City, UT, United States

**Keywords:** primary cilia, adipose tissue, adipocyte progenitor cells, diabetes, signaling, ciliopathies, beige adipocyte, thermogenesis

## Abstract

The primary cilium is a cellular sensory organelle found in most cells in our body. This includes adipocyte progenitor cells in our adipose tissue, a complex organ involved in energy storage, endocrine signaling, and thermogenesis. Numerous studies have shown that the primary cilium plays a critical role in directing the cell fate of adipocyte progenitor cells in multiple adipose tissue types. Accordingly, diseases with dysfunctional cilia called ciliopathies have a broad range of clinical manifestations, including obesity and diabetes. This review summarizes our current understanding of how the primary cilium regulates adipocyte progenitor cell fate in multiple contexts and illustrates the importance of the primary cilium in regulating energy storage and adipose tissue function.

## Introduction

The primary cilium is a solitary cellular protrusion that functions like an antenna and is highly conserved in eukaryotic evolution (Klena and Pigino, 2022). In the human body, most cells are ciliated, including stem and progenitor cells (Yanardag and Pugacheva, 2021). Consistent with its near-ubiquitous presence, studies over the past two decades have shown that primary cilia on different cells and in different tissues serve distinct functions, including sensing light, odorants, mechanical stimulation, and chemical ligands (Ishikawa and Marshall, 2011). This functional breadth is achieved *via* the expression of cell context-specific ciliary receptors and downstream signaling components (Hilgendorf et al., 2016; Wachten and Mick, 2021).

The basal body, composed of the mother centriole and pericentriolar material, nucleates the primary cilium and is anchored to the plasma membrane. The primary cilium consists of a microtubule-based axoneme that is surrounded by a ciliary membrane, which contains the ciliary receptors to enable its specific sensory function (Nachury and Mick, 2019). The length of the primary cilium can differ dramatically depending on cell type, ranging from 2 to 10 µm in length. Ciliary morphology is a function of ciliary protein composition and is hence controlled by the trafficking of ciliary cargo (Mukhopadhyay et al., 2008; Kuhns et al., 2019). Microtubule motor proteins couple to the intraflagellar transport machinery (IFT) to traffic ciliary proteins, and this process is tightly regulated (Hibbard et al., 2022).

Genetic mutations affecting ciliary structure or ciliary protein composition cause a heterogeneous group of human genetic disorders called ciliopathies (Hildebrandt et al., 2011). A subset of human ciliopathies, such as Bardet-Biedl Syndrome (BBS, OMIM #209900) and Alström Syndrome (ALMS, OMIM #203800), are associated with obesity and metabolic disorder (Hilgendorf, 2021). Several recent studies have highlighted the importance of the primary cilium in regulating whole-body energy homeostasis. This is accomplished by ciliary signaling in multiple cell types and in multiple tissues with relevance to metabolism (see accompanying reviews in this edition). This review summarizes our current understanding of how the primary cilium regulates energy storage in adipose tissue.

### Adipose tissue types and functions

Fat in the body is stored in adipose tissue, a complex organ that is composed of myriad cell types (Lenz et al., 2020). Across the body, there are many adipose tissue depots. Primarily, these are classified into visceral depots located within the body cavity and subcutaneous depots found under the skin (de Jong et al., 2015). These classifications are further broken down into distinct fat pads with variable roles.

Most of the body’s fat is found in the energy-storing white adipose tissue (WAT). In addition to energy storage, these WAT depots are institutional in endocrine signaling through the sensing and secretion of hormones, lipids, and metabolites (Lehr et al., 2012; Cinti, 2018). WAT is highly abundant and found in close association with many organs and in a multitude of functionally distinct visceral and subcutaneous depots (de Jong et al., 2015; Zwick et al., 2018). Perhaps most notable are the subcutaneous inguinal depot lining the skin of the abdomen just above the thigh and the perigonadal visceral depot surrounding the gonads within the body cavity; both of these are present in humans and mice and have been commonly studied for their varied differentiation capacity and ease of identification and access (Chusyd et al., 2016). By volume, WAT is nearly 90% composed of terminally differentiated white adipocytes (Yoshimura et al., 2009). White adipocytes are energy-storing cells that utilize a singular, unilocular lipid droplet that fills the majority of the cell. White adipocytes are capable of increasing their total stored energy *via* the expansion of this lipid droplet well after their differentiation (Konige et al., 2014).

Brown adipose tissue (BAT) performs adaptive heat generation through a process known as thermogenesis. These depots were originally thought to be found only in infants, but recent studies reveal a more functional role in adults (Cypess and Kahn, 2010). BAT is primarily found in subcutaneous depots in close proximity to muscle around the shoulder blades and neck. Additional, albeit minor, visceral BAT depots can be found along the kidneys and around the aorta (de Jong et al., 2015). Brown adipocytes express high levels of the mitochondrial-associated protein UCP1, which is essential for thermogenesis. In contrast to white adipocytes, these cells contain multilocular lipid droplets broken up by an extensive network of mitochondria (Chechi et al., 2013).

Another emerging population of adipocytes are classified as beige adipocytes, which have also been referred to as “brite”, “paucilocular”, “inducible brown”, or “recruitable brown” adipocytes (Frontini and Cinti, 2010; Schulz et al., 2011; Cinti, 2012; Contreras et al., 2014; Townsend and Tseng, 2015). As their name suggests, these adipocytes display characteristics typical of both white and brown adipocytes (Paulo and Wang, 2019). Residing in WAT depots, beige adipocytes are stimulated by beta-adrenergic or cold shock stimuli; after which, they express higher levels of UCP1, their lipid droplet becomes multilocular, and they have increased thermogenic capacity, though to a much lesser degree than a true brown adipocyte (Nedergaard and Cannon, 2013). After stimulation, they can revert to a white adipocyte-like state with the potential to transdifferentiate upon subsequent restimulation (Rosenwald et al., 2013). It has been argued that human brown adipocytes in adults are more similar to beige adipocytes than classical brown adipocytes, though this is still a source of debate (Sharp et al., 2012; Chen et al., 2019; Cannon et al., 2020). Due to brown and beige adipocytes’ propensity to burn stored fat, utilization of beige adipocyte expansion within WAT, or “beiging”, has been investigated to combat weight gain that contributes to the growing obesity epidemic (Alcala et al., 2019; Rabiee, 2020).

Expansion of WAT is possible through two mechanisms: hypertrophy or hyperplasia. In hypertrophy, preexisting mature adipocytes accumulate triglycerides within their lipid droplets, thus expanding their overall size. Alternatively, hyperplasia is resultant of new adipocytes differentiating within the fat pad from adipocyte progenitor cells (APCs) (Rosen and Spiegelman, 2006). Lineage tracing techniques in mice have recently demonstrated the variable expansion of adipose tissue from different depots in response to different stimuli (Wang et al., 2013; Jeffery et al., 2015; Jeffery et al., 2016; Gao et al., 2018). These studies showed that visceral fat depot expansion in male mice was both hypertrophic and hyperplastic, while inguinal expansion was near exclusively hypertrophic. Conversely, it was demonstrated that female mice exhibit perigonadal and inguinal WAT hyperplasia, highlighting sexual dimorphic patterning of fat depot expansion (Jeffery et al., 2016). In fact, fat distribution and expansion vary greatly between individuals due to hormonal signals and genetic factors (Davis et al., 2013; Karastergiou and Fried, 2017; Zwick et al., 2018).

Not all fat storage is the same, as visceral expansion of WAT is associated with increased insulin intolerance compared to inguinal expansion (Lee et al., 2013; Karpe and Pinnick, 2015; Vishvanath and Gupta, 2019). While obesity can result from both hyperplastic and hypertrophic expansion, hyperplastic expansion is more metabolically healthy and less likely to increase the chances of developing diabetes or other metabolic or obesity-related diseases (Carey et al., 1997; Misra et al., 1997; Wang et al., 2005; Sanchez-Gurmaches and Guertin, 2014a). An emerging model to explain the consequences of hypertrophic fat expansion proposes that both the inability to develop new adipocytes and the hypertrophic nature of existing adipocytes overcrowds WAT, leading to hypoxia and cell death (Jo et al., 2010; Longo et al., 2019). Adipocytes under elevated levels of stress from high nutrient storage may undergo apoptosis, and concurrent with lipodystrophies, the relocation of their stored lipids may lead to harmful inflammation and fibrosis (Fischer-Posovszky et al., 2011; Davis et al., 2013; Trouwborst et al., 2018).

### Adipocyte progenitor cells

#### APCs in white adipose tissue

The cellular composition of WAT depots varies greatly. Collectively, the non-adipocyte cells in each depot are referred to as the stromal vascular fraction (SVF), consisting of fibroblasts, nerve cells, vascular cells, and immune cells. Far outnumbering any other cell type in the SVF and often outnumbering mature adipocytes are APCs, also referred to as “pre-adipocytes” (Tchkonia et al., 2007; Baker et al., 2017). Like mature adipocytes, APCs have dynamic roles in adipose tissue, namely that of distilling multiple signals from their surroundings to induce or delay their differentiation into mature adipocytes.

Conversion from an APC to a mature adipocyte is marked by increased PPARγ transcription factor expression in response to signals like insulin after caloric intake (Wang et al., 2013; Jeffery et al., 2015). Dubbed a master regulator of adipogenesis, this nuclear receptor is both necessary and sufficient to drive white adipocyte differentiation (Tontonoz et al., 1994; Rosen et al., 1999). Much of our current understanding of adipogenesis at the molecular level derives from the murine fibroblast 3T3-L1 pre-adipocyte cell line. These cells have been used as *in vitro* models of APCs and their differentiation can be achieved upon treatment with insulin, the glucocorticoid dexamethasone, and the cyclic AMP-increasing drug 3-isobutyl-1-methylxanthine (IBMX) (Rosen et al., 2000; Tang et al., 2008).

**FIGURE 1:**
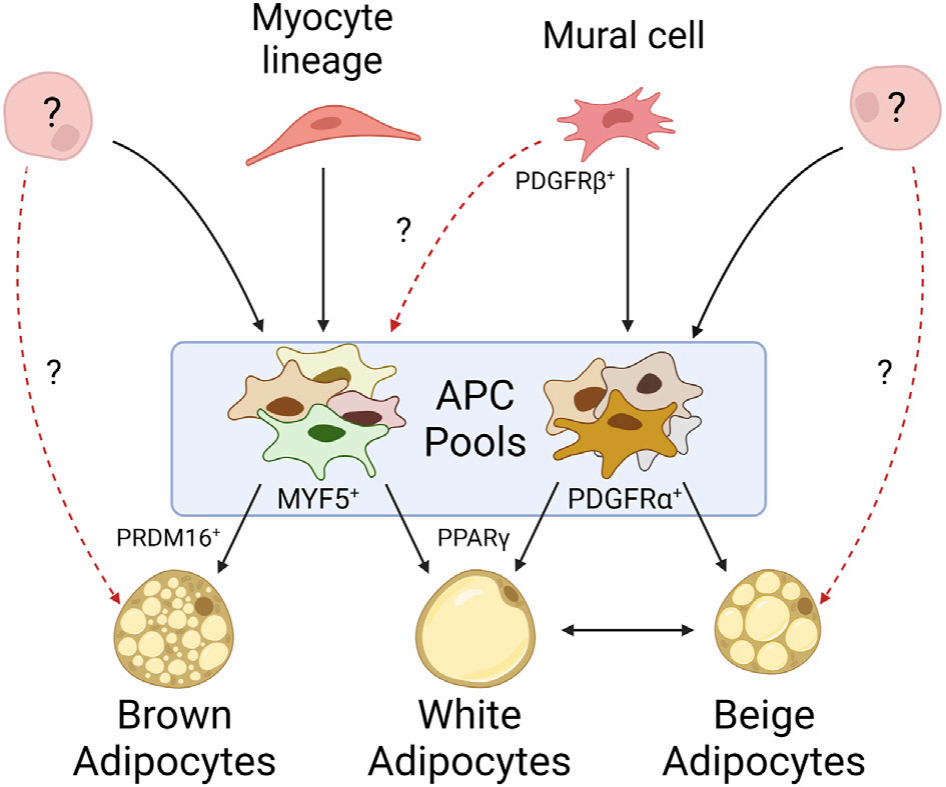
Mature adipocytes arise from heterogeneous progenitor pools. Mural cells give rise to many of the white APCs identified within WAT depots, but their propensity to give rise to brown adipocytes is unknown. APCs derived from myocyte lineages typically differentiate into brown adipocytes, though myocyte markers have been identified on APCs within WAT. While it is known APCs can arise from alternative cell lineages, it is unclear to what extent they contribute to adipose tissue. Beige adipocytes exemplify our lack of understanding as their origin may be derived from an exclusive progenitor, solely through white adipocyte transdifferentiation, or some combination of both.

Murine APCs can be isolated by fluorescent activated cell sorting (FACS) using Lin- (CD45^−^ CD31^−^ TER119-) CD34^+^ CD29^+^ SCA1+ markers. These cells can be differentiated into mature adipocytes *ex vivo* and can reconstitute WAT depots in lipodystrophic mice (Rodeheffer et al., 2008). However, this sorting strategy yields a heterogenous population of cells, and the different APC subpopulations display variable adipogenic capacity, expression profiles, and cell surface markers (Prunet-Marcassus et al., 2006; Joe et al., 2009; Baglioni et al., 2012; Macotela et al., 2012). Platelet derived growth factor receptor alpha (PDGFRα) is a key identifier of APCs, but not mature adipocytes, in all significant WAT depots, however its level of expression is variable (Lee et al., 2012; Berry and Rodeheffer, 2013). Additional markers have been shown to be specific to certain WAT depots, highlighting the heterogeneity of APC subpopulations (Figure 1) (Joe et al., 2009; Macotela et al., 2012).

Lineage tracing has allowed insight into the origins of these subpopulations. Specifically, genetic labeling of cells expressing PDGFRβ, a canonical mural cell marker, identified mural cells with enriched adipocyte marker expression in inguinal WAT depots but failed to identify these cells in other (non-WAT) tissues. Conversely, lineage tracing of PPARγ, which marks committed adipocyte lineages, only identified a subset of mural cells from inguinal WAT (Tang et al., 2008). Together, these data suggest committed adipocytes arise from mural cells, but only a subset of mural cells within WAT commit to the adipocyte lineage. Other studies measuring GFP expression driven by the promoter of an APC-associated transcription factor, ZFP423, observed labeling of both mural and endothelial cells in inguinal WAT and BAT depots; however, a follow-up study demonstrated that endothelial-derived APCs have little contribution to overall adipose tissue development (Kanda et al., 2009; Gupta et al., 2010; Gupta et al., 2012; Tran et al., 2012). Additional studies highlight alternative origins for APCs. Cells derived from the neuronal crest seem to give rise to a specific subset of APCs found in the cranium and face (Billon et al., 2007). Further PPARγ lineage tracing found committed APCs migrated to inguinal WAT depots during embryonic development or shortly after birth, suggesting early APC pools may be composed of cells from developmentally distinct origins (Tang et al., 2008; Zwick et al., 2018). Altogether, these lineage tracing studies demonstrate great diversity in APC origins (Figure 1).

Similarly, single-cell RNA sequencing studies describe distinct subpopulations of APCs within and between WAT depots, each with variable differentiation capacities (Church et al., 2014). Subpopulations across depots can share common functions while preserving surface marker expression and transcriptional profiles more representative of their depot of origin (Burl et al., 2018). Fibro-inflammatory progenitor (FIP) subpopulations identified in the perigonadal WAT can be inhibitory to adipogenesis and do not express PPARγ (Hepler et al., 2018; Schwalie et al., 2018; Spallanzani et al., 2019). Highly proliferative interstitial progenitor cells (IPCs) in inguinal WAT share similar transcription profiles to FIPs but will differentiate *in vitro* while FIPs will not. These IPCs are capable of differentiating into multiple cell fates other than just adipocytes. Additional APC subpopulations express high concentrations of PPARγ and represent a committed adipocyte lineage, while others express non-canonical adipocyte cell surface markers but are still capable of adipogenesis *ex vivo* (Merrick et al., 2019). Similarly, studies on human adipose tissue corroborate the complex heterogenous environment of cells within WAT. Single-cell sequencing from a limited cohort identified six populations of human APCs all expressing PDGFRα (Emont et al., 2022). These progenitor populations were shown to be functionally similar to the previously identified subpopulations of committed, inhibitory, and pluripotent murine APCs described above (Hepler et al., 2018; Schwalie et al., 2018; Merrick et al., 2019). Notably, Emont et al. (2022) also elucidated that human APC subpopulations, similar to murine, demonstrate preferential depot specificity. Further non-transcriptional characteristics of these subpopulations, such as ciliation status, have not been rigorously assessed (Marion et al., 2009; Hilgendorf et al., 2019). However, it should be noted multiple studies implicate the importance of primary cilia in the differentiation of both murine and human APCs into mature adipocytes, with ciliary defects having detrimental effects on adipogenesis and metabolic health (Forcioli-Conti et al., 2015; Hilgendorf et al., 2019; Peraldi et al., 2020). This will be further discussed below.

#### APCs in brown adipose tissue

BAT shares an expression profile more similar to myogenic tissue than to WAT, possibly reflective of its role in energy expenditure and high mitochondrial content (Timmons et al., 2007). The myocyte markers MYF5 and PAX3 appear to be surface expression markers present on most brown APCs, suggesting a progenitor distinct from those of WAT, and instead derived from skeletal muscle (Figure 1) (Seale et al., 2008). However, not all brown adipocytes trace to MYF5+ or PAX3+ populations, such as those observed by Sanchez-Gurmaches and Guertin in the perirenal and peri-aortic depots (Sanchez-Gurmaches and Guertin, 2014b). Thus, this suggests an alternative, unidentified progenitor(s) capable of maturing into brown adipocytes. Additionally, MYF5+ and PAX3+ populations have been identified in WAT depots as well, particularly those near BAT such as the anterior subcutaneous and retroperitoneal depots. This heterogeneity of APCs within BAT may be reflective of functional heterogeneity among mature brown adipocytes as they do not all share the same thermogenic activity (Song et al., 2020). In fact, lower mitochondrial content and decreased UCP1 expression is observed in brown adipocytes that accumulate larger (less multilocular) lipid droplets, more akin to their white adipocyte energy-storing cousins. *In vitro* studies have shown expression of the transcription factor PRDM16 in both differentiating and differentiated immortalized and *ex vivo* brown APC cultures. Its expression is sufficient to differentiate myoblasts into brown adipocytes. Its deletion *in vivo*, however, has shown that PRDM16 is not necessary for brown adipocyte differentiation, but rather results in the accumulation of white adipocytes in BAT (Seale et al., 2007; Harms et al., 2014). These studies highlight the importance of PRDM16 in brown APC cell fate and differentiation. While differentiating brown APCs have been shown to be ciliated, the ciliation status of all brown APC subpopulations, and the importance of cilia during brown adipocyte differentiation, has been underexplored (Nosavanh et al., 2015).

#### The origin of beige adipocytes

Unlike white and brown adipocytes, beige adipocytes are not confined to their own depots. Beige adipocytes can be found in WAT in response to cold shock stimuli or beta-adrenergic signaling (Xue et al., 2005). There are two common models to explain this beiging within WAT: the first suggests beige adipocytes arise from the transdifferentiation of mature white adipocytes, while the second suggests they originate from a distinct subpopulation of APCs in WAT (Figure 1) (Cinti, 2012; Wu et al., 2012; Cinti, 2018). Not all APCs can become beige adipocytes and not all depots are prone to beiging, suggesting beiging is both cell- and depot-specific (Cinti, 2009; Frontini and Cinti, 2010; Cinti, 2011; Walden et al., 2012). Lineage tracing suggests beige adipocytes are not represented by a single lineage, as different WAT depots harbor beige adipocytes derived from different APC subpopulations (Lee et al., 2012; Sanchez-Gurmaches et al., 2012; Wu et al., 2012). In support of the transdifferentiation model, UCP1 labeling indicates that after beiging, an inguinal adipocyte can functionally return to its native “white” state, as well as be restimulated into a beige adipocyte (Rosenwald et al., 2013). However, it is unclear if this transdifferentiation potential is specific to a distinct subset of adipocytes. These data support the contribution of both models to the presence of beige adipocytes in WAT. Beige APCs also share similarities to brown APCs, including MYF5+ subpopulations (Sanchez-Gurmaches et al., 2012). Similar to PRDM16’s role in brown adipocytes, it also plays a role in the expression of UCP1 in beige adipocytes (Wang et al., 2019a). The role of beiging in human adipose tissue is still largely underexplored, though there have been characterized differences between the propensity of similar depots across species to undergo beiging (Zuriaga et al., 2017).

### Ciliary signaling pathways regulating adipose tissue

**FIGURE 2:**
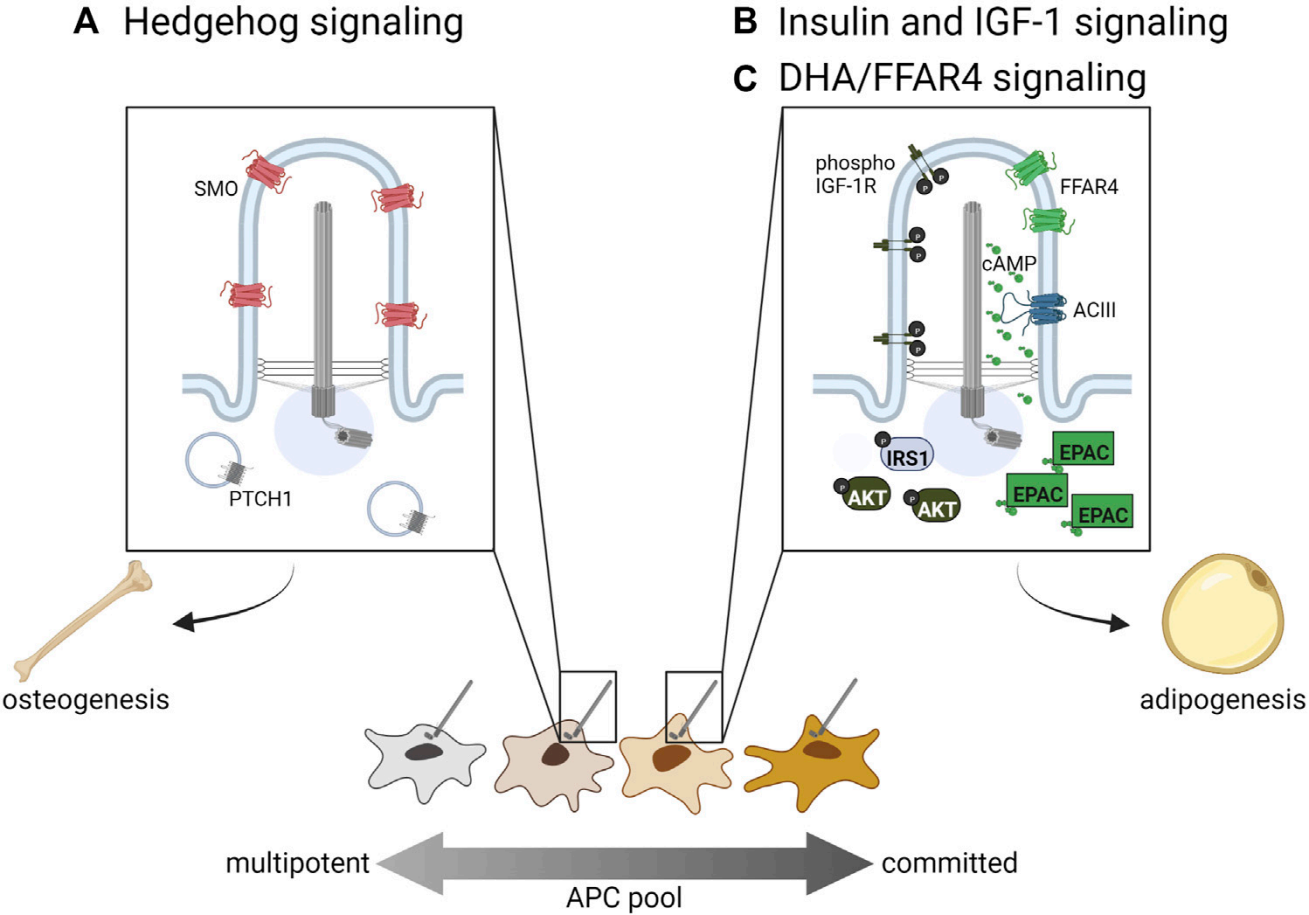
Ciliary signaling pathways regulating APC fate. **(A)** Hedgehog signaling inhibits adipogenic lineage commitment and hence adipogenesis, while **(B)** insulin, IGF-1R, and **(C)** omega-3 fatty acids such as DHA promote adipogenesis *via* primary cilia-localized receptors. Distinct APC subpopulations may display differential sensitivity to these ciliary signals directing cell fate.

APCs are the only ciliated cells described in adipose tissue to date. White, beige, and brown mature adipocytes, which are not ciliated, arise from ciliated APCs in both humans and mice (Marion et al., 2009; Forcioli-Conti et al., 2015; Nosavanh et al., 2015; Hilgendorf et al., 2019). The primary cilium functions like an antenna, with both tissue- and cell-specific receptors that appropriately sense extracellular ligands and direct differentiation. The prevalence and length of APC cilia vary by depot, species, age, and sex. Human APC cilia dynamically change during adipogenic differentiation, and this may be due to ciliary trafficking of different receptors (Dalbay et al., 2015; Forcioli-Conti et al., 2015). It has also been shown that obese-derived human APCs have shorter, dysfunctional cilia in comparison to lean-derived counterparts (Ritter et al., 2018a; Ritter et al., 2018b). Some of these differences in cilia length and prevalence may be due to the heterogeneity of APC subpopulations described above (Figure 1). To date, APC cilia morphology and prevalence have not been systematically studied in different adipose tissue contexts. Because cilia morphology can change due to ciliary cargo trafficking, studying how these changes relate to signaling function in different contexts is an important future direction for the field. A number of ciliary signaling pathways regulating APC fate have been described, and three major pathways are summarized below (Figure 2).

#### Hedgehog signaling in white and brown APCs

Hedgehog (Hh) signaling regulates the development and maintenance of specific tissue types in all bilaterians by directing cell fate. In vertebrates, it acts specifically in primary cilia through the trafficking of two main receptors, PTCH1 and SMO. When Hh signaling is activated, PTCH1 is trafficked out of the cilium while SMO accumulates in the cilium and promotes the generation of the activator form of GLI transcription factors, which then promote transcription of target genes (for a recent comprehensive review on Hh signaling in primary cilia see (Kopinke et al., 2021)). In WAT, Hh signaling increases the commitment of multipotent stem cells towards osteogenesis, thereby inhibiting adipogenesis by reducing the number of committed APCs (Figure 2A). *In vitro*, the use of sonic Hh on the multipotent cell lines C3H10T1/2 and ST2 resulted in increased osteoblast commitment (Spinella-Jaegle et al., 2001). Hh activation inhibits adipogenesis in multipotent NIH-3T3 and C3H10T1/2 cells; in the pre-adipocyte line 3T3-L1 it not only inhibits adipogenesis, but induces expression of osteogenic markers, suggesting Hh can alter cell fate even in more committed APCs (Suh et al., 2006). Suh et al. has also shown in *Drosophila* that fat-body specific Hh activation inhibits fat formation, while fat-body Hh inhibition promotes fly adiposity. Similarly, a mouse model with constitutively active Hh signaling due to a mutation in PTCH1 has reduced WAT compared to controls (Li et al., 2008).

Hedgehog signaling also inhibits the formation of BAT. Hh pathway activation in mouse embryos through PTCH1 deletion or constitutively active SMO results in reduced BAT formation, where BAT is replaced by cells resembling skeletal-muscle progenitors (Nosavanh et al., 2015). Addition of SMO agonist (SAG) to brown APCs *in vitro* inhibits differentiation, while this effect is reversed with treatment of the Hh inhibitor cyclopamine (Nosavanh et al., 2015). In a SUFU knockout mouse model, WAT is significantly lost while BAT remains at normal amounts, which may be due to the dual role of SUFU in regulating both the activator and repressor forms of GLI (Pospisilik et al., 2010; Liu et al., 2012). Hh signaling can also regulate metabolism of WAT and BAT *in vitro* through a non-canonical SMO/AMPK signaling axis that increases aerobic glycolysis. This metabolic effect is induced in both 3T3-L1 and primary brown adipocytes upon either SAG or cyclopamine treatment; it was determined to be a primary cilia-dependent response, as there was no observed effect in cilia ablated IFT88 or KIF3A knockout murine embryonic fibroblasts (Teperino et al., 2012). Taken together, local Hh pathway activation inhibits adipose tissue formation by directing progenitor cell fate away from adipogenesis and instead towards osteogenesis.

#### Insulin and IGF-1 signaling promote adipogenesis *via* primary cilia

Insulin and Insulin-like Growth Factor One (IGF-1) are both important regulators of WAT and BAT (Boucher et al., 2016). Both of these molecules stimulate adipogenesis by binding to their receptors, insulin receptor and IGF-1R (Smith et al., 1988; Wabitsch et al., 1995; Entingh-Pearsall and Kahn, 2004). They can bind each other’s receptors as well, although with a reduced affinity (Smith et al., 1988; Entingh-Pearsall and Kahn, 2004). Ligand binding to these receptors stimulates tyrosine kinase activity, which results in phosphorylation of insulin receptor substrate proteins (IRS 1–4), recruitment and activation of PI3-kinase, and ultimately activation of AKT/protein kinase B, which is required for adipogenesis (Zhang et al., 2009). In APCs, IGF-1R is more highly expressed than insulin receptor, though insulin receptor expression increases during differentiation into non-ciliated adipocytes (Smith et al., 1988; Back and Arnqvist, 2009). In 3T3-L1 cells, IGF-1R localizes to both the primary cilium and the plasma membrane, but ciliary IGF-1R is more sensitive to insulin stimulation, resulting in the recruitment and activation of downstream signaling components IRS-1 and AKT at the ciliary base (Figure 2B, left side of primary cilium) (Zhu et al., 2009). Zhu et al. also show that ablation of primary cilia using *Ift88* or *Kif3a* knockdown causes decreased activation of AKT and reduced adipogenesis. In congruity, Yamakawa et al. (2021) find that primary cilia regulate adipogenesis by controlling the accumulation of IGF-1R and downstream signaling components in Caveolin-1 positive lipid rafts around the ciliary base.

While IGF-1R evidently participates in ciliary signaling, it is unclear if insulin receptor, having two isoforms A and B, also localizes to the cilia of APCs. While insulin receptor isoform B is expressed in mature adipocytes, insulin receptor isoform A is expressed in APCs (Back and Arnqvist, 2009). Isoform A has been shown to localize to primary cilia of pancreatic β-cells (Gerdes et al., 2014). Therefore, it is plausible that both IGF-1R and insulin receptor isoform A localize to APC cilia to promote adipogenesis. Importantly, IGF-1R and insulin receptor regulate both WAT and BAT development, with double knockout mice showing almost a complete absence of WAT and BAT (Boucher et al., 2016). Separately, IGF-1R knockout mice have decreased WAT and BAT, while insulin receptor knockout mice have almost no WAT but increased BAT with unilocular lipid droplets. It is uncertain if IGF-1 or insulin signaling regulates the differentiation of brown adipocytes in a ciliary manner.

#### Ciliary FFAR4 promotes adipogenesis

Free fatty acid receptor four (FFAR4), also known as G-protein coupled receptor 120 (GPR120), is a member of a family of five G-protein coupled receptors that are activated by free fatty acids (Hirasawa et al., 2005). FFAR4 can localize to the plasma membrane of cells, including mature adipocytes, where it stimulates glucose uptake; it has also been shown to localize to the primary cilium of APCs and pancreatic islet cells (Oh et al., 2010; Hilgendorf et al., 2019; Wu et al., 2021). FFAR4 is specifically activated by dietary omega-3 fatty acids, including docosahexaenoic acid (DHA), and its activation in cilia stimulates ciliary cAMP signaling (Figure 2C, right side of primary cilium). Like insulin and IGF-1, cAMP is an important regulator of adipogenesis, as illustrated by the inclusion of the phosphodiesterase inhibitor IBMX in *ex vivo* adipogenesis-inducing cocktails. Although vital for adipocyte differentiation, it was unclear what factors might stimulate cAMP signaling during *in vivo* adipogenesis. FFAR4 has been shown to localize to the primary cilium of 3T3-L1 pre-adipocytes, isolated murine and human APCs, and murine APCs *in vivo* (Hilgendorf et al., 2019). FFAR4 is trafficked to the cilium in a TULP3-dependent manner, and its activation in 3T3-L1 cells results in ciliary cAMP signaling. FFAR4-induced adipogenesis requires the cAMP effector protein EPAC, while Protein Kinase A is dispensable. Treating 3T3-L1 cells with the FFAR4 ligand DHA promotes downstream expression of adipogenic genes such as CEBPα *via* chromatin remodeling (Hilgendorf et al., 2019). Therefore, ciliary cAMP can trigger adipogenesis upon activation of ciliary FFAR4, linking dietary omega-3 fatty acids to adipogenic cAMP signaling. Ciliary FFAR4 also acts in pancreatic α-cells and β-cells, where its activation stimulates ciliary cAMP signaling and promotes glucose-stimulated glucagon and insulin secretion, respectively (Wu et al., 2021). Thus, the primary cilium plays a critical role in adipose tissue by regulating APC commitment through ciliary Hh signaling and triggering APC differentiation through ciliary IGF-1 receptor and FFAR4 signaling.

### Changes to adipose tissue in patients and mouse models with altered cilia

**FIGURE 3:**
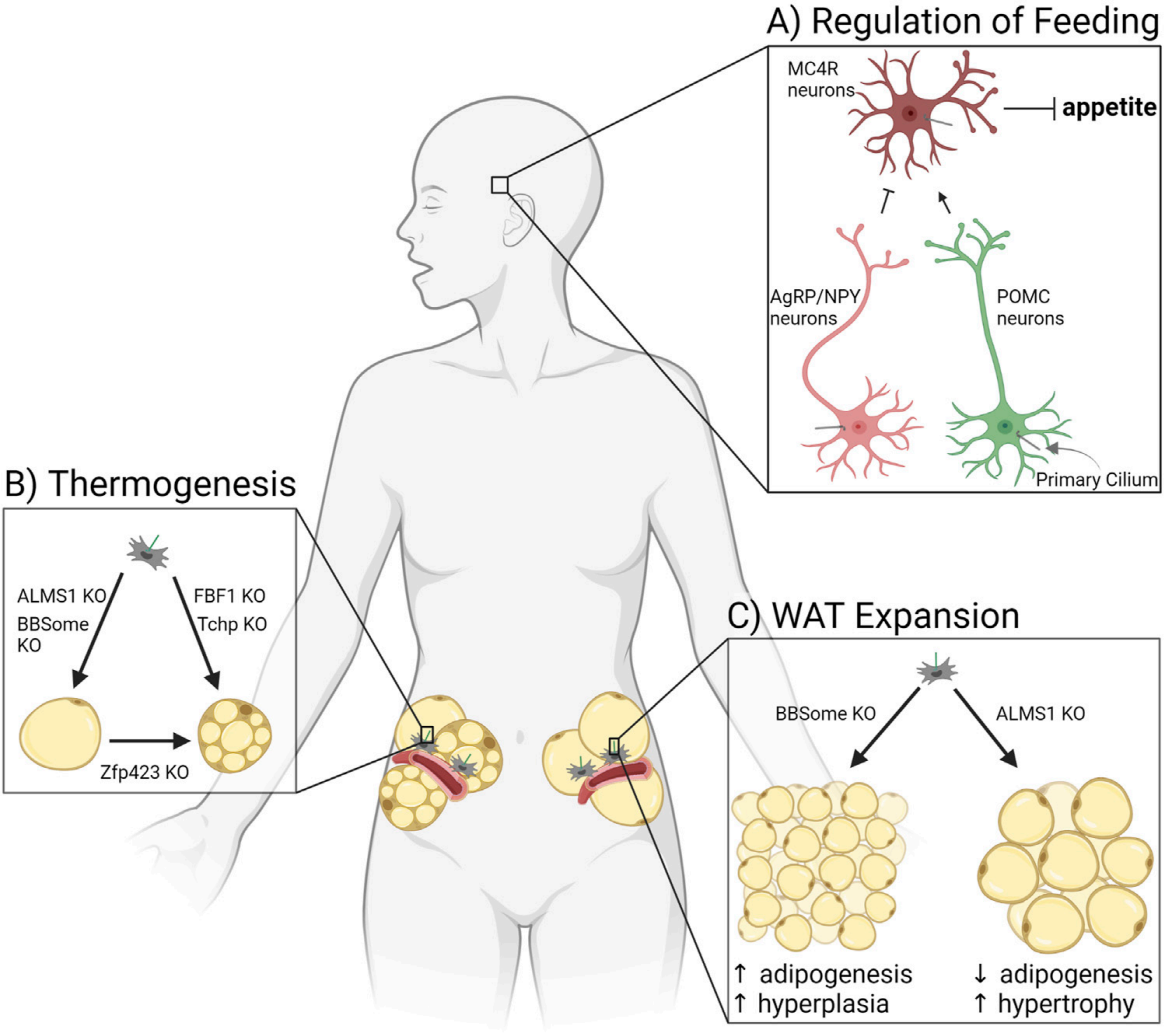
Ciliary signaling regulates adipose tissue. **(A)** Primary cilia of three types of neurons in the hypothalamus regulate appetite, thereby controlling adiposity due to hyperphagia. **(B)** Adipocyte potential for thermogenesis in white adipose tissue is affected by ciliary protein knockouts (KO). **(C)** Expansion of white adipose tissue through adipogenesis is controlled by ciliary signaling pathways altered in patients and mice deficient for the BBSome or ALMS1.

Whole body metabolism and energy storage are affected by multiple ciliary signaling pathways functioning in APCs, as well as ciliary signaling in other tissues (Figure 3). This variety of possible signaling effectors complicates the analysis of ciliary mouse models as exemplified by BBS and ALMS patients. Both diseases are caused by alterations to ciliary signaling in different ciliated cell types and result in obesity, but BBS patients are relatively metabolically healthy, while ALMS patients are metabolically unhealthy. Below we summarize how changes to cilia affect energy storage *via* satiety regulation, thermogenesis, and white adipocyte morphology.

#### Ciliary dysfunction and hyperphagia-driven obesity

Patients with BBS are characterized by many symptoms, with one major condition being obesity (Beales et al., 1999). BBS is caused by mutations in one of 22 genes that help form or are associated with the BBSome, a protein complex required for trafficking a subset of GPCRs out of the primary cilium (Nachury et al., 2007). Mouse models of BBS are also obese. In pair-feeding experiments, where food consumption was matched to control mice, the body weight of BBS knockout mice was rescued, arguing that BBS knockout-induced obesity is due to hyperphagia (Rahmouni et al., 2008).

The arcuate nucleus of the hypothalamus is a central regulator of food intake and satiety sensing in the brain (Figure 3A). Neurons that synthesize agouti-related protein (AgRP) and neuropeptide Y (NPY) function to increase appetite and decrease energy expenditure, while pro-opiomelanocortin (POMC) neurons suppress feeding and enhance energy expenditure (Varela and Horvath, 2012). Peripheral organs secrete hormones such as leptin, insulin, and ghrelin based on the energy status of the body. These circulating hormones cross the blood-brain barrier to activate or inhibit AgRP/NPY neurons or POMC neurons. These neurons in turn secrete factors that activate or inhibit a third type of hypothalamic neuron, the melanocortin-4 receptor (MC4R) expressing neurons in the paraventricular nucleus of the hypothalamus. MC4R neurons regulate appetite and energy expenditure (Baldini and Phelan, 2019). All three of the discussed neuron types in the hypothalamus are ciliated, and loss of cilia or ciliary receptors results in hyperphagia-driven obesity. Of note, MC4R is the most commonly mutated gene in monogenic morbid obesity, and patients with these mutations are hyperphagic (Farooqi et al., 2003). MC4R localizes to primary cilia of MC4R neurons, and obesity-associated mutations impair its ciliary localization (Siljee et al., 2018). Similarly, type III adenylyl cyclase (ACIII/AC3) knockout mice are obese due to hyperphagia (Wang et al., 2009). AC3 also localizes to primary cilia of neurons, and several AC3 polymorphisms are strongly associated with obesity in humans (Bishop et al., 2007; Nordman et al., 2008). Mouse models lacking cilia (through IFT88 or KIF3A depletion) are obese due to hyperphagia and show adipocyte hypertrophy, decreased BAT function, and increased levels of leptin and insulin, in addition to glucose, insulin, and leptin resistance (Davenport et al., 2007; Sun et al., 2021). There are numerous receptors that localize to the primary cilium of AgRP/NPY and POMC neurons to control food intake (for a recent list of ciliary receptors implicated in feeding regulation see (Hilgendorf, 2021; Wachten and Mick, 2021)). Receptors for the satiety hormone leptin have been found in cilia of mature olfactory neurons (Baly et al., 2007). BBS knockout mice have abnormal leptin receptor trafficking and signaling, suggesting that leptin may signal through primary cilia in hypothalamic neurons as well (Seo et al., 2009). Primary cilia also play a role in neuron development. A mouse model with hypomorphism of ciliary gene RPGRIP1L shows an altered ratio of AgRP/NPY neurons to POMC neurons, leading to hyperphagia and obesity in mice (Stratigopoulos et al., 2014; Wang et al., 2019b). Thus, primary cilia in hypothalamic neurons have a substantial effect on satiety sensing and hence adipose tissue. For an in-depth discussion of this ciliary function, see the accompanying review by Dr. Nicolas Berbari in this edition.

#### Ciliary dysfunction and thermogenesis

Thermogenesis occurs in brown or beige adipocytes, for the purpose of both thermal regulation and energy balance in the body. Recent mouse model studies link primary cilia to the control of adipose tissue’s potential for thermogenesis (Figure 3B). Fas binding factor one (FBF1) is a ciliary transition fiber component, and FBF1 knockout mice are obese without metabolic dysfunction while also demonstrating beiging in both their subcutaneous and visceral WAT depots (Zhang et al., 2021). Differentiating primary murine or human APCs that lack FBF1 induces a brown adipocyte-like morphology, with increased mitochondria and expression of UCP1. Similarly, trichoplein keratin filament binding (TCHP) knockout mice are resistant to high fat diet-induced obesity, have smaller adipocytes, maintain an increased temperature, and show upregulation of UCP1, suggesting a thermogenic program in WAT (Yamakawa et al., 2021). Mutations in ZFP423 induce a ciliopathy phenotype (Hong and Hamilton, 2016), and as discussed above, ZFP423 has been shown to play a critical role in both white and brown adipocyte differentiation (Gupta et al., 2010). However, mouse models in which ZFP423 is depleted in mature (unciliated) adipocytes also show beige-like adipocytes and upregulation of thermogenic genes in subcutaneous and visceral WAT (Shao et al., 2016). These beige adipocytes were shown to be derived from white adipocytes, not APCs, suggesting additional non-ciliary roles for ZFP423. Thermogenesis can also be inhibited by ciliary changes. A BBS4 knockout in mice results in defective thermogenesis, including an impaired beiging of subcutaneous WAT and lower maintainable body temperature under cold exposure (Gohlke et al., 2021). Likewise, ALMS1 mutant mice have defective adaptive thermogenesis in response to HFD and cold exposure compared to wildtype littermates (Poekes et al., 2017). Primary cilia can control thermogenic programs in adipose tissue, and this area of research will likely grow due to interest in beiging as a potential way to treat obesity and metabolic dysfunction.

#### Ciliary dysfunction and WAT composition: hypertrophy *vs*. hyperplasia

As described earlier, WAT expands in response to caloric excess by both hypertrophy (strongly associated with being metabolically unhealthy) and hyperplasia (expansion considered metabolically healthy). Primary cilia in adipose tissue have a substantial effect on this expansion balance because cilia control the ability of the tissue to make new adipocytes, and therefore participate in hyperplastic expansion (Figure 3C). This is done through signaling pathways described above, such as *via* FFAR4 and insulin/IGF-1 receptors (Figures 2B, C). In a mouse model where the primary cilium was specifically removed in APCs using a *Pdgfrα*-driver, neither adult males nor females expanded their WAT compared to littermate controls or were able to generate new adipocytes (Hilgendorf et al., 2019). Likewise, ablation of the whole primary cilium *via* IFT88 or KIF3A depletion in 3T3-L1 pre-adipocytes caused a significant differentiation defect (Zhu et al., 2009; Hilgendorf et al., 2019). For more information on APC dysfunction and obesity see (Louwen et al., 2018).

ALMS is a ciliopathy caused by mutations in the ALMS1 gene, which is thought to regulate ciliary trafficking and hence ciliary signaling (Alvarez-Satta et al., 2021). Patients with ALMS are obese with extreme insulin resistance that is disproportionate to body weight and adiposity (Minton et al., 2006; Han et al., 2018; Tahani et al., 2020). The subcutaneous WAT of ALMS patients shows increased adipocyte hypertrophy compared to body mass index (BMI)-matched control subjects (Geberhiwot et al., 2021). Similarly, mouse models of ALMS are obese and insulin resistant due to adipose tissue dysfunction and adipocyte hypertrophy (Geberhiwot et al., 2021). Depletion of ALMS1 in 3T3-L1 cells impairs adipogenesis (Huang-Doran and Semple, 2010), consistent with the increase in adipocyte hypertrophy observed in ALMS patients and mouse models. Re-expression of wild-type ALMS1 in fetal APCs and mature adipocytes in ALMS mutant mice completely rescued obesity, adipocyte hypertrophy, and insulin sensitivity (Geberhiwot et al., 2021). In contrast, patients with BBS are obese due to hyperphagia but are metabolically healthier than BMI-matched control subjects (Beales et al., 1999). The subcutaneous WAT of BBS patients shows smaller adipocytes compared to BMI-matched control subjects, and the depletion of BBSome components in isolated human APCs promotes adipogenesis, consistent with increased hyperplastic WAT expansion (Marion et al., 2009; Marion et al., 2012). Taken together, these studies argue that ALMS1-mediated ciliary trafficking is required for adipogenesis while BBSome-mediated ciliary trafficking inhibits adipogenesis. Further studying ciliary regulation of adipogenesis, and thus the balance between hyperplastic and hypertrophic expansion, will help us understand these diseases and how APC cilia could promote metabolically healthy forms of energy storage.

## Conclusion and Future Challenges

The primary cilium has emerged as a central regulator of metabolism, functioning in multiple tissues in the body and multiple cell types within a tissue. To date, most of the research has focused on the role of hypothalamic neuron primary cilia in regulating feeding. Recently, a number of researchers have highlighted the importance of the primary cilium to energy homeostasis in peripheral organs, including in the pancreas (see the accompanying review by Dr. Jing Hughes in this edition) (Volta et al., 2021; Wu et al., 2021; Cho et al., 2022), muscle (Palla et al., 2022), and adipose tissue (Hilgendorf et al., 2019; Yamakawa et al., 2021). Here, we discussed the importance of the primary cilium in regulating energy storage in adipose tissue, focusing primarily on ciliary signaling pathways organized by the primary cilium of APCs.

Recent single-cell transcriptomic and lineage tracing studies have revealed that our current definition of APCs encompasses a highly heterogeneous group of cells. Importantly, these distinct APC subpopulations are functionally linked to differences in adipogenic capacity and even cell fate. We do not yet know if there are differences in the percent ciliation, ciliary morphology, or ciliary receptor composition among APC subpopulations within or between adipose tissue depots. We propose that the primary cilium plays a critical role in informing the adipogenic capacity and cell fate of distinct APC subpopulations. Hence, we predict that there will be measurable and important differences in ciliary morphology and ciliary receptor composition among different APC subpopulations.

While the list of ciliary receptors is ever-growing, to date only a select few ciliary signaling pathways have been described in APCs. We hypothesize that many more ciliary receptors localize to distinct APC subpopulations and are functionally important for differentiation. Discovering the identity and function of these receptors is an active area of investigation, and recent advances in single cell transcriptomics and proximity proteomics have accelerated this research. Moreover, we do not yet know how all these ciliary signaling pathways intersect to direct cell fate. Does activation of one pathway sensitize or inhibit another ciliary pathway, and if yes, what is the mechanism? Does this ciliary remodeling change the adipogenic capacity of APCs, and is this change transient or permanent?

Finally, there are multiple pathways that regulate the trafficking of ciliary receptors into and out of the primary cilium. In APC cilia, ALMS1 appears to traffic ciliary cargo that is required for adipogenesis, while the BBSome traffics ciliary cargo that inhibits adipogenesis. This argues that the ciliary receptor composition of APC primary cilia is not only functionally important, but that it can be regulated. Further research is required to identify targets that can increase metabolically healthy, hyperplastic WAT expansion or beiging of WAT by changing the ciliary receptor composition and hence modulating the adipogenic capacity of APCs.
